# The South Texas Medico-Chirurgical Society

**Published:** 1902-06

**Authors:** 


### The South Texas Medico-Chirurgical Society.

The South Texas Medical Society held its regular meeting at Beaumont on the 4th and 5th inst., Dr. R. T. Morris, of Houston, President, and Dr. D. S. Weir, Secretary.

